# High-Throughput Measurements of Stem Characteristics to Estimate Ear Density and Above-Ground Biomass

**DOI:** 10.34133/2019/4820305

**Published:** 2019-05-30

**Authors:** Xiuliang Jin, Simon Madec, Dan Dutartre, Benoit de Solan, Alexis Comar, Frédéric Baret

**Affiliations:** ^1^INRA EMMAH, UMR 1114 228 route de l'Aérodrome, 84914 Avignon, France; ^2^Institute of Crop Sciences, Chinese Academy of Agricultural Sciences/Key Laboratory of Crop Physiology and Ecology, Ministry of Agriculture, Beijing 100081, China; ^3^HIPHEN, Rue Charrue, 84000 Avignon, France; ^4^ARVALIS-Institut du Végétal, Station Expérimentale, 91720 Boigneville, France

## Abstract

Total above-ground biomass at harvest and ear density are two important traits that characterize wheat genotypes. Two experiments were carried out in two different sites where several genotypes were grown under contrasted irrigation and nitrogen treatments. A high spatial resolution RGB camera was used to capture the residual stems standing straight after the cutting by the combine machine during harvest. It provided a ground spatial resolution better than 0.2 mm. A Faster Regional Convolutional Neural Network (Faster-RCNN) deep-learning model was first trained to identify the stems cross section. Results showed that the identification provided precision and recall close to 95%. Further, the balance between precision and recall allowed getting accurate estimates of the stem density with a relative RMSE close to 7% and robustness across the two experimental sites. The estimated stem density was also compared with the ear density measured in the field with traditional methods. A very high correlation was found with almost no bias, indicating that the stem density could be a good proxy of the ear density. The heritability/repeatability evaluated over 16 genotypes in one of the two experiments was slightly higher (80%) than that of the ear density (78%). The diameter of each stem was computed from the profile of gray values in the extracts of the stem cross section. Results show that the stem diameters follow a gamma distribution over each microplot with an average diameter close to 2.0 mm. Finally, the biovolume computed as the product of the average stem diameter, the stem density, and plant height is closely related to the above-ground biomass at harvest with a relative RMSE of 6%. Possible limitations of the findings and future applications are finally discussed.

## 1. Introduction

Ear density (the numbers of ears per m^2^) is generally well correlated with above-ground biomass and grain yield at maturity of wheat [1, 2]. However, the correlation may depend on environmental conditions as well as genotypes. Most stems observed at harvest bear an ear: stem density (the number of stems per m^2^) appears thus as a good proxy of the ear density [3]. Stem density depends thus both on plant density and on the number of stems per plant which is quantified by the tillering coefficient. The environmental conditions experienced by the crop and the genotype control the tillering coefficient [4]. Therefore, several studies report the interest of the ear and stem density as traits to be used in the selection process of wheat genotypes [4, 5]. Further, plant height and stem diameter are highly correlated with the above-ground biomass in wheat [6–10]. Therefore, stem density, ear density, plant height, and stem diameter are thus highly desired to score the performances of a genotype in wheat crop breeding programs.

The number of stems per plant is difficult to evaluate when plants start to produce tillers since plants are often intricated and hardly identifiable. Further, the number of stems per plant may change with time due to possible tiller regression during tillering and stem elongation stages. After the flowering stage, most stems bear an ear and the stem density therefore provides a good proxy of the ear density. Ear and stem densities are therefore usually measured at maturity by manual counting over a given sample area. The stem diameter is rarely measured since it is very tedious and time consuming. Similarly, above-ground biomass is rarely measured extensively for the same reasons. Crop height at harvest is most frequently measured in the field using a ruler. In addition to the limits of these low-throughput invasive measurements that require large human resources to be completed, the small sampling area used and errors associated with the manual measurements may result in significant uncertainties on these variables that would limit the repeatability and heritability as computed from the experimental observations. It appears therefore necessary to develop new methods for accurate measurements of the stem density, crop height, and stem diameter for wheat crops within large field phenotyping experiments.

The recent advances in high-resolution imaging systems and computing capacity as well as image processing algorithms offer great opportunities to develop nondestructive high-throughput methods. Jin et al. [11] and Liu et al. [12] have demonstrated that the plant density could be estimated at early stages in wheat crops from high-resolution imagery. Direct estimates of the tillering coefficient at the end of the tillering stage were investigated by several authors with application to the management of nitrogen fertilization for stable crops. Vegetation indices computed from the reflectance measurements have been empirically related to the tiller density [13–15]. However, reflectance measurements are mainly sensitive to the amount of green foliage, which is loosely related to the stem density. Alternatively, several authors have developed algorithms for estimating wheat stem density at early stages from high-resolution imagery [16]. Unfortunately, this method, applied to plants in pots grown under greenhouse conditions, is difficult to transfer to field conditions. Further, the number of stems at relatively early stages may overestimate the actual stem density at harvest because of possible tiller regression as already pointed out. Previous scientists have used algorithms for estimating wheat ear density in-field conditions using RGB or thermal imagery [17–20]. However, these techniques, operated from the top of the canopy before harvest, may be limited when a significant number of ears are laying in the lower layers of the canopy. Previous studies have also demonstrated that above-ground biomass (AGB) can be estimated using different remote sensing platforms [21–27]. However, the correlative nature of these relationships questioned their robustness when applied outside the domain where they have been calibrated.

The aim of this study is to develop and evaluate a method to estimate stem density after the harvest. Images of the remaining stems cut by the combine machine during harvest show a clear circular cross section at their tip that could be identified by machine vision techniques. Further, the diameter of the stem could be also measured to tentatively estimate the AGB by combining the average stem diameter with the stem density and plant height. High-throughput estimates of plant height have become now a standard trait easy to compute from 3D point clouds derived from LiDAR or standard cameras aboard drone [28]. The main objectives of this study are therefore (1) to develop a method for identifying stems from postharvest submillimetric RGB images and compute the stem density; (2) to compare the estimated stem density with the ear density measured with traditional invasive methods; (3) to estimate the stem diameter and describe their distribution; and (4) to investigate the capacity of stem density, stem diameter, and plant height to provide a proxy of AGB. The field experiments and data acquisition are first described. The developed methods are then presented, and their performances to estimate stem density, stem diameter, and AGB are finally evaluated and discussed.

## 2. Materials and Methods

### 2.1. Experimental Sites and Ground Measurements

The Gréoux and Clermont sites located in France (Table 1) were hosting wheat phenotyping experiments with about one thousand microplots of 13 rows by 10 m length (Gréoux) or 8 rows by 2.5 m length (Clermont). For both sites, rows were spaced by 17.0 cm. A subsample of microplots (Table 1) was selected in both sites for the development and validation of the method. They included genotypes with contrasted tillering capacity and plant architecture as well as variation in irrigation (Gréoux) and nitrogen (Clermont) crop management.

The ear density (ears/m^2^) was measured in the field at maturity for the 66 (Gréoux) or 123 (Clermont) microplots considered, by counting the ears over three samples of two rows by 1.0 m length corresponding to a 1.02 m^2^ sampled area. The AGB (g/m^2^) was measured in Gréoux over 37 microplots by collecting all the plants within three samples of two rows by 1.0 m length. The samples were then oven-dried at 70°C for three days and finally weighed. The height (cm) of the plants was measured using two LMS400 LiDARs (SICK, Germany) fixed on a phenomobile, i.e., a robot rover that automatically moved in the field and collected the measurements. More details on height measurements are given by Madec et al. in [28].

### 2.2. Image Acquisition and Visual Labeling of Stems

A Canon EOS 550D RGB camera with a resolution of 5184 by 3456 pixels equipped with a 29 mm focal lens was fixed on a pole and maintained at a 1.2 m distance from the ground at the Gréoux experimental site. The camera was set to speed priority. The same operating mode was used in Clermont, except that the camera was Sony ILCE-6000 with 6000 by 4000 pixels equipped with a 60 mm focal length lens and maintained at 1.8 m from the ground. The images were recorded in JPG format on the SD memory card. Measurements were completed under cloudy illumination conditions with light wind. Three (Gréoux) or four (Clermont) images were taken over each microplot. A subsample corresponding to four rows by 0.6 m length for Gréoux and four rows by 1.6 m length for Clermont (Table 2) was extracted in the center of each image. This offered the advantage of minimizing image deformation observed mostly on the borders of the whole image. The Gréoux images were first resampled using a bicubic interpolation algorithm to provide the same resolution as that of Clermont.

**Figure 1:**
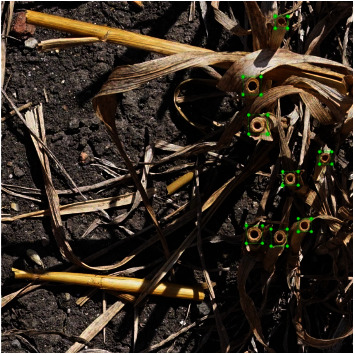
Visual stem identification. Each stem identified corresponds to a green bounding box. Note: the image is actually cropped from original image by 1000x1000 pixel.

The good quality of images provided strong confidence in the visual identification of the stems (Figure 1). A bounding box was interactively drawn around each stem identified in the images. The bounding box used to identify each stem was designed to include enough elements surrounding the stem (Figure 1). A total of 822 images were visually annotated to be used for the calibration and validation of the method.

### 2.3. Object Detection Using Faster-RCNN

Convolutional Neural Networks (CNNs) are powerful machine learning methods [29]. They are widely used to extract imagery information features and then classify objects. CNNs were trained using large collections of diverse images to extract more effectively rich feature representations. These CNNs features often outperform handcrafted ones such as histogram of oriented gradients (HOG), local binary patterns, or speeded up robust features [30]. The TensorFlow (https://www.tensorflow.org/) implementation of Faster Regional Convolutional Neural Network (Faster-RCNN) by the object detection application programming interface (API) [31] was achieved here. Faster-RCNN has been widely used to detect objects [32]. The region proposal network (RPN) branch was inserted between the conv4 and conv5 blocks. The Inception-Resnet-V2 model was used as it obtained the best accuracy among several modern object detectors [31]. An anchor was set at each location considered by the convolution maps of the RPN layer. Each anchor was associated with a size and aspect ratio. A set of 12 anchors with different size and aspect ratio were assigned at each location, following the default setting. The number of proposed regions per patches was set to 300, which was consistent with the expected number of stems per patch. Note that the images used here were 1000 x 1000 pixels since the memory requirements were too demanding for larger images. The original images were thus split into 1000 x 1000 patches, keeping 50% overlap between neighboring patches to minimize possible problems associated with the borders. The batch size was fixed to 1 and the threshold value for the non-maxima suppression with an IOU (Intersection Over Union) was set to 0.2. The model was trained with a learning rate of 0.0003 and a momentum of 0.90. The model was first pretrained on the Common Objects in Context (COCO) dataset to provide the starting point. The COCO dataset [31] contains 0.33 million images with 1.5 million of object instances belonging to 80 object categories. More details on the Faster-RCNN used could be found in Madec et al. [20]. The pretrained model was then fine-tuned over the calibration image extracts. It identified and localized stems using a bounding box associated with a confidence score varying between 0.0 and 1.0.

**Figure 2:**
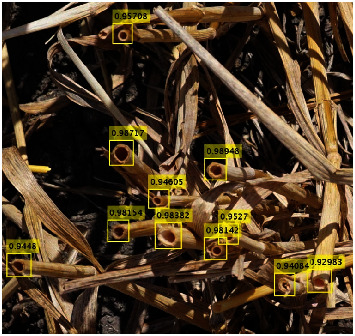
Application of the stem detection using Faster-RCNN algorithm to an image extract in Gréoux. Each yellow bounding box corresponds to the identified stem and is associated with its score corresponding to the probability of containing a stem.

The trained model was finally applied to all the image extracts available. When identified stem bounding boxes were overlapping, a minimum of 0.75 overlap fraction was used to eliminate one of the overlapping bounding boxes. Finally, bounding boxes with a confidence score value smaller than 0.80 were not considered as stems. This score threshold value was optimized to get the best stem density estimation performances. An example of the Faster-RCNN stem detection result is presented in Figure 2. The estimated stem density (stem/m^2^) was eventually computed by dividing the number of stems identified over the image extracts of a microplot by the size of the extracts (Table 2).

### 2.4. Estimating the Stem Diameter and Biovolume

**Figure 3:**
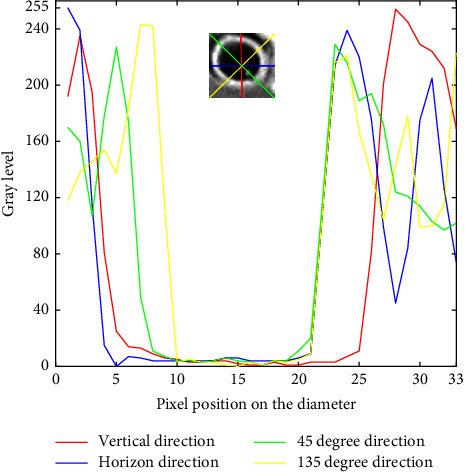
The extraction of stem diameter of each sub-window image using diameter gray level profile.

The bounding box of the identified stems was first transformed into gray images using the value (V) component of the HSV transform [33]: V=0.2989R+0.5870G+0.1140B, where R, G, and B were, respectively, the red, green, and blue components of the RGB images coded in 8 bits. The gray value profiles were then extracted along four compass directions: 0°, 45°, 90°, and 135° (Figure 3). The gray level profiles show typical patterns with high values corresponding to the border of the stem and lower values outside and inside the stem (Figure 3). The two borders of the stem were thus identified using the two maximum gray values. The distance between the maximums values was computed and then averaged over the four compass directions to provide an estimate of the diameter.

The stem diameter was used to compute the area of the section of the stem. The basal area of each microplot was then computed as the average area of the stem section multiplied by the stem density. Finally, the biovolume was computed as the product of the basal area and the plant height as derived from the LiDAR measurements.

### 2.5. Statistical Analysis

Both Gréoux and Clermont datasets were randomly split into 2/3 for model calibration and 1/3 for validation. A first global training (called here Cgc) was investigated by pooling the calibration datasets of Gréoux and Clermont sites. The same was done for the validation datasets Vgc. The performances of this global calibration (Cgc) were also evaluated on both the Gréoux (Vg) and Clermont (Vc) validation datasets. Then, a cross-validation was also investigated to better evaluate the robustness of the classification: the calibration was completed on the Gréoux (Cg) or Clermont (Cc) calibration datasets and validated on the Gréoux (Vg) and Clermont (Vc) validation datasets.  Table 3 presents the several cases considered.

A detected stem bounding box (i.e., with a score >0.8) was considered correct (true positive, TP) if its IOU with a labeled stem bounding box was larger than the IOU threshold value. Otherwise, the detected stem bounding box was considered as false positive (FP). The proposed bounding boxes with a score<0.8 (i.e., not considered as stems) with IOU larger than the IOU threshold value were considered false negative (FN). The IOU threshold value was set to the usual value of 0.5. The precision (TP/(FP+TP)), recall (TP/(FN+TP)), and bias (1-(precision/recall)) were also calculated.

### 2.6. Heritability Computation

The broad sense heritability (H^2^) evaluates the repeatability of the stem or ear density estimates. It was computed as the percentage of the genotypic variance, Vg, to the total variance, Vg+Ve, where Ve is the variance due to the environment [34]. The heritability of the stem density and ear density was computed over sixteen wheat genotypes (122 plots) selected from the Clermont experimental site where each genotype was replicated six to fifteen times.

## 3. Results and Discussion

### 3.1. Stems Are Accurately Identified Using the Faster-RCNN Model

To evaluate the robustness of the RCNN model, it was calibrated on the Gréoux (Cg), Clermont (Cc), or both datasets (Cgc). Performances computed over the validation datasets were very good with 0.91 <* precision*< 0.96 and 0.93 <* recall* < 0.97 (Table 4). Precision and recall were well balanced with a small bias: -0.03 <* bia*s<0.01.

The results showed that the classification accuracy of stem identification was very high based on the precision and recall values over the same experiments (Table 4). The robustness of the classification was further investigated by comparing the precision and recall values computed over the validation datasets coming from the other experiments. Results show that the classification evaluated over the same experiment used to calibrate the model was always performing the best (Table 4). The classification performances decreased significantly when the model calibrated over a single experiment was validated on the other experiment. This may be explained both by the limited sample size of the calibration dataset and also by the specific features associated with each experiment, including the spatial resolution (Tables 2 and 3). However, when the calibration was completed over the pooled experiments (Cgc), the precision and recall values decreased only slightly when evaluated over each individual experiment (Vg or Vc) (Table 5). The model captured the key information common to the two experiments to provide a consistent stem identification. It confirmed the efficiency and robustness of the Faster-RCNN method.

### 3.2. Stem Density Is Accurately Estimated

The consequences of the identification performances of the Faster-RCNN model discussed previously were evaluated in terms of plant density at the image extract level. For the sake of consistency, several calibration and validation datasets were considered to further evaluate the robustness of the model. Results showed RRMSE values ranging from 6.08% to 9.19%. Calibrating over the pooled datasets (Cgc, Table 5) provided the best performances with RRMSE lower than 7%. A slight degradation of the performances was observed when calibrating over a single dataset. Calibrating over the Gréoux dataset provided the worst performances when validated over the Clermont dataset (RRMSE=9.2%) because of the smaller sample size and variation in the cutting height and inclination of the stems during harvest between Gréoux and Clermont sites. In the following, we have used the Faster-RCNN trained over the pooled Gréoux and Clermont calibration datasets (Cgc) that provided more robust performances.

**Figure 4:**
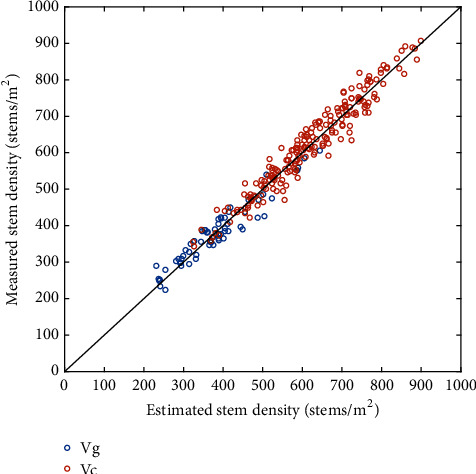
Comparison between the stem density estimated using Faster-RCNN method calibrated over the pooled Cgc dataset and the stem density evaluated visually over the images. The black line corresponds to the 1:1 line; the red and blue circles correspond, respectively, to the Gréoux (Vg) and Clermont (Vc) validation datasets.

When considering the calibration over the pooled dataset (Cgc) that provided the overall best performances, very small biases were observed with points closely distributed around the 1:1 line (Figure 4). The scatter around the 1:1 line appeared to be relatively independent of the stem density (Figure 4).

### 3.3. The Stem Density Is a Good Proxy of the Ear Density

**Figure 5:**
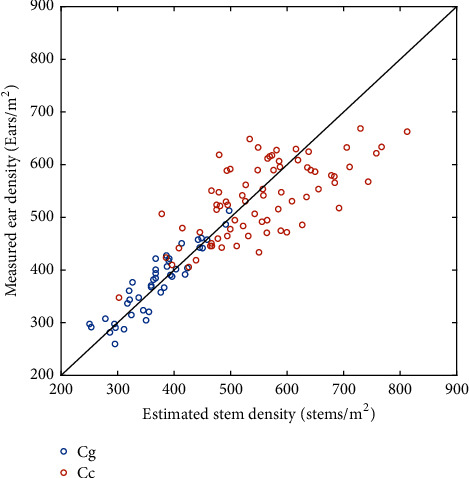
Relationship between the estimated stem density and the measured ear density at the Gréoux (blue dots) and Clermont (red dots) datasets. The black line corresponds to the 1:1 line.

The stem density estimated with the Faster-RCNN model calibrated over the Cgc dataset was compared to the ear counted visually at the ground level. Both quantities were evaluated on different samples, expected however to represent the average microplot value. Results showed that the estimated stem density based on the Faster-RCNN model was very consistent (Figure 5) with the measured ear density at the Gréoux (Table 6) and Clermont (Table 6) experimental sites. The scatter between ear and stem densities appeared to increase with the density: this was obvious between the Gréoux (250<density<550) and Clermont (400<density<800) sites. Part of the larger scatter observed over the Clermont site might come from a smaller sample size for the ear density visual counting (1.02 m^2^). The scatter between ear and stem densities seems to increase with the density within the Clermont site between the low and high densities (Figure 5). Nevertheless, the good agreement found between ear and stem densities was thus confirming the results of Siddique et al. [3].

Previous studies demonstrated that the RGB imagery can be used to estimate ear density using image processing algorithms [17–20]. However, ear density estimation performances were generally limited to a comparison between the ears detected by the machine learning algorithm and those that can be visually identified by and operated on the image. Some discrepancies could appear compared with the actual ear density, particularly when some ears are lying in the lower canopy layers and could not be easily seen from the top of the canopy. Counting ears from the stem sections appears therefore preferable under such conditions. Further, stem sections are relatively simpler objects to identify as compared to ears that may show a large aspect variability. Additionally, ears can frequently overlap in the field, making their identification more complex as compared to stem sections that never overlap.

### 3.4. Stem and Ear Densities Are Highly Heritable

The heritability (H^2^) values of the stem density and ear density were compared at the Clermont experimental site where several replicates of 16 genotypes were available. Results show that the H^2^ values of stem density (80.1%) and ear density (78.3%) were high and close together. This is consistent with the strong relationship found between both quantities (Figure 5). These heritability values agreed well with the values provided by Madec et al. (2019) [20]. The H^2^ value of the stem density was slightly higher than that of the ear density, probably because of the larger sample size used for estimating the stem density from the RGB images, which makes the values more repeatable. The high values of heritability found suggested that the proposed method will be well suited to serve the breeders needs.

### 3.5. Stem Diameter Follows a Gamma Distribution

The distribution of stem diameter was investigated at Gréoux and Clermont experimental sites, respectively, on 66 and 156 microplots. The distribution of the stem diameter may be a pertinent trait describing the structure of the tiller population that may be impacted by the growth conditions. The distribution of the stem diameter of each microplot was adjusted either to a normal or to a gamma distribution. The corresponding p values associated with the fit of each distribution was computed. Results show that the p value of the gamma distribution was larger than that of the normal distribution for 80% of the microplots for Gréoux and 84% of the microplots for Clermont. The gamma distribution characterized by a scale and a shape parameter was therefore selected to describe the stem diameter distribution over each microplot.

**Figure 6:**
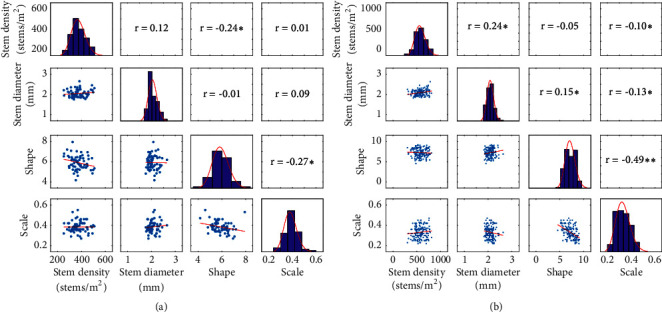
Correlation and distribution between stem density, average stem diameter, shape, and scale parameters over Gréoux (a) and Clermont (b) experimental sites. The correlation coefficient,* r*, is given in the upper triangular matrix with *∗∗* and *∗* corresponding, respectively, to significant values at 0.01 and 0.05 probability levels.

The average stem diameter of each microplot ranged from 1.8 to 2.5 mm, with a median value close to 2.0 mm for both sites (Figure 6). The average stem diameter was loosely but positively correlated to the stem density (Figure 6): the stress experienced by the plants was affecting both the density and the diameter of the stems, with no apparent compensations between these two traits. The stem diameter distribution for each microplot as described by a gamma function was further investigated: shape parameters were slightly smaller for the Gréoux site (4<shape<8) as compared to those of the Clermont site (5<shape<10). Conversely, scale parameters were slightly larger for the Gréoux site (0.3<scale<0.6) as compared to the Clermont site (0.2<scale<0.5). The distribution of the diameters was more concentrated around the average for the Clermont site as compared to the Gréoux site where a larger range of diameters was observed. This may be related to the stress conditions that were stronger in Gréoux, particularly during the stem elongation phase. This was also reflected by the stem density that was more impacted in Gréoux. The scale and shape parameters were negatively correlated for both sites, with a stronger correlation for Clermont (Figure 6). Since the average of a gamma distribution is defined by the product of the shape and scale parameters, the negative correlation between the two parameters was explained by the constraint to keep the average close to 2.0 mm. Therefore, both parameters could be equally used to describe the “flatness” of the stem diameter distribution.

### 3.6. The Biovolume Is a Good Proxy of the Above-Ground Biomass

**Figure 7:**
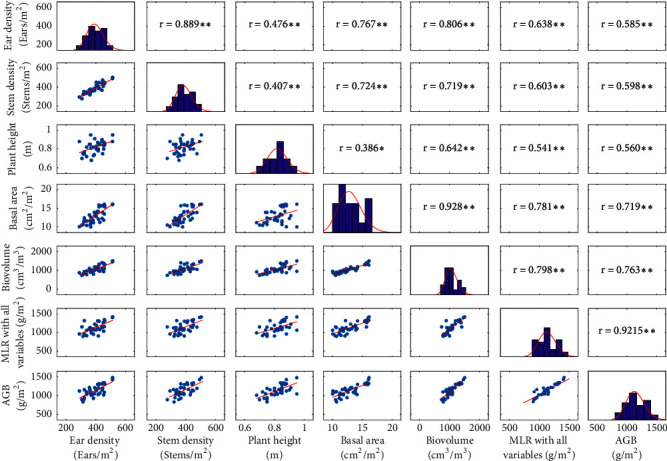
Correlation matrix between the AGB and the six variables investigated. Note: *∗∗* and *∗* mean correlation significant at the 0.01 and 0.05 level of probability, respectively.

A total of 37 microplots from the Gréoux dataset were used to relate the measured AGB with the ear density and the four structural traits derived from high-throughput measurements: stem density, stem basal area computed as the product of the average stem diameter and the stem density, plant height, and biovolume computed as the product of the basal area and plant height. Results show that all these traits are strongly correlated with AGB (Figure 7 and Table 7). The best relationship is however obtained using the biovolume that combines the three main original traits: stem density and average stem diameter that are combined into the basal area; and plant height. Note that these traits are relatively independent: stem density and plant height are loosely correlated (Figure 6, r^2^=0.16); plant height and basal area are also loosely correlated (r^2^=0.15).

Because the dataset used was limited, the predictive performances of the relationships observed between the AGB and the five traits were evaluated using a leave-one-out cross-validation method (Jin et al., 2018). The best determination coefficients were observed consistently for the biovolume (Table 7) with a relative error of 5.8%, i.e., within the order of magnitude of the accuracy with which AGB was measured. Our results are very consistent with those presented by Aziz et al. (2004) and Pittman et al. (2015).

To further improve the predictive model, we used all the five traits together within a multiple linear regression model. Marginal improvement of the model performances was observed (Figure 7 and Table 7). This may be explained by the strong relationships between the five traits used, as well as the decrease in the degree of freedom induced by the increase of the number of coefficients to be adjusted (six coefficients instead of two needed when using only the biovolume). The biovolume appeared therefore as a very sound proxy of the AGB. Previous results suggested that AGB could be estimated using different optical techniques and technologies [21–27]. Our study further confirmed these results. The results demonstrated that the estimation accuracy of AGB could be improved by combining LiDAR data and RGB imagery. However, the stability of the relationship found over the limited sample used in this study should be further evaluated with emphasis on the possible dependency on the environmental and management conditions, as well as on differences between genotypes.

## 4. Conclusion

This study demonstrated that the identification of the stems after the harvest was possible using deep-learning approaches applied to RGB images. This requires the spatial resolution to be sufficient, i.e., around 0.2 mm since the stem diameters are around 2.0 mm. It ensures that the objects to be identified within the image are represented with an optimal number of pixels ranging between 40 and 120 pixels as advised by Madec et al. [20]. Such a high resolution could be achieved using a high-resolution RGB camera fixed either on a pole, on a cart, on a phenomobile, or even on a UAV flying at low altitude as already demonstrated by Jin et al. [11]. Alternatively, a set of RGB cameras could be mounted on the combine machine and provide, in near real time, an estimate of the stem density.

The method requires the stems not to be covered by the straw rejected by the combine machine. Further, too inclined stems due to the harvest process or some postharvest practice may result in degraded performances since the sections of the tip of the stems will not be viewed by the camera or will be strongly deformed. Further, the proposed method may be not suitable under stem lodging situations where the stem sections will show unexpected patterns. Nevertheless, the objects to be identified are relatively simple, which would indicate that the Faster-RCNN model trained over the data used in this study would be robust. Changes in the illumination conditions may have little impact of the stem identification since the objects are mostly identified by the relative brightness of the pixels, with the color itself bringing very little information. We demonstrated therefore that the stem density is accessible with high-throughput, relatively low cost and with a very good accuracy. Further, the capacity to sample large area to estimate the stem density will minimize the impact of the spatial variability within a microplot.

Although Madec et al. [20] among others demonstrated that similar deep-learning techniques could be applied efficiently to estimate the ear density, ear identification is more complex because of strong differences of the ear aspect between cultivars and environment, as well as possible occlusion of some ears by the top ears or the top leaves. We demonstrated in this study that the stem density was a very close proxy of the ear density although some discrepancy is expected under specific environmental conditions. In such circumstances, the distribution of the diameter of the stems could potentially provide the necessary information to get a better estimate of the ear density from the stem density and diameter distribution.

Once the stem is identified, we demonstrated that the diameter could be easily measured. The distribution of the stem diameters followed a gamma function with an average diameter close to 2.0 mm. The distribution of the stem diameters may be indicative of the structure of the tiller population that may be governed by the genetics in interaction with the sowing density and pattern as well as the environmental conditions experienced by the plants. Finally, the biovolume computed as the product of the average stem diameter, the stem density, and plant height was demonstrated to be a close proxy of the above-ground biomass. This opens very attractive potential for the breeders to get high-throughput estimates of the total plant biomass at harvest and possibly quantify the radiation use efficiency and the harvest index assuming that the yield will be measured anyway. Nevertheless, these promising results should be verified under a much larger number of situations to verify that the correlations are not too dependent on the environmental conditions as well as on the genotype.

## Figures and Tables

**Table 1:** Characteristics of the Gréoux and Clermont experimental sites.

Sites	Latitude	Longitude	Number of plots	Sowing date	Sowing density (seeds/m^2^)
Gréoux	43°45′N	5°53′E	66	27/10/2015	300
Clermont	45°46′N	3°70′E	123	02/11/2016	280

**Table 2:** Characteristics of the images taken over the two experimental sites.

Sites	Date of images	Distance to ground (m)	Ground resolution (mm)	Sampled area (m^2^)
Gréoux	12/07/2016	1.20	0.18	1.23
Clermont	18/07/2017	1.80	0.12	4.36

**Table 3:** Characteristics of the data sets used for the calibration and validation of the algorithm. Statistics of the stem density are indicated for each data set, including minimum (Min), mean (Mean), maximum (Max), range (Range), standard deviation (SD), and coefficient of variation (CV) of the stem density.

	Number of image extracts				Stem density (stem/m^2^)				
Dataset	Name	Gréoux	Clermont	Min	Mean	Max	Range	SD	CV (%)
Calibration	Cgc	132	416	112	493	991	879	132	26.77
Validation	Vgc	66	208	161	561	906	745	179	31.91

Calibration	Cg	132		112	357	605	493	77	21.57
Validation	Vg	66		161	352	601	440	78	22.16

Calibration	Cc		416	308	549	991	683	101	18.04
Validation	Vc		208	373	662	906	533	116	17.52

**Table 4:** Accuracy of stem identification using the Faster-RCNN method. Results are presented for three calibration datasets (Cg, Cc, and Cgc). The evaluation is achieved on the validation datasets (Vg, Vc, Vgc).

Calibration dataset	Validation dataset	Precision	Recall	Bias
Cg	Vg	0.95	0.97	-0.02
	Vc	0.91	0.93	-0.02
	Vgc	0.92	0.94	-0.02

Cc	Vg	0.92	0.95	-0.03
	Vc	0.96	0.95	0.01
	Vgc	0.96	0.95	0.01

Cgc	Vg	0.94	0.95	-0.01
	Vc	0.96	0.95	0.01
	Vgc	0.95	0.95	0.00

**Table 5:** Performances of the stem density estimation when using Faster-RCNN method for the postclassification step. The evaluation is achieved on the three validation data sets (Vg, Vc, and Vgc).

Calibration dataset	Validation dataset	Sample size	slope	intercept	R^2^	RMSE (stems/m^2^)	RRMSE (%)
Cg	Vg	66	0.98	25.63	0.95	24.45	6.95
	Vc	208	0.94	41.25	0.85	60.82	9.19
	Vgc	274	0.95	38.12	0.90	40.23	7.17

Cc	Vg	66	0.95	20.85	0.88	30.52	8.67
	Vc	208	0.97	17.14	0.96	40.25	6.08
	Vgc	274	0.96	30.28	0.92	39.66	7.07

Cgc	Vg	66	0.95	33.12	0.91	25.46	7.23
	Vc	208	0.97	45.33	0.92	42.81	6.47
	Vgc	274	0.98	38.16	0.94	38.45	6.85

**Table 6:** Statistics of the relationships between the estimated stem density and the measured ear density. The Faster-RCNN was trained over the Cg+Cc dataset.

Datasets	Slope	Intercept	R^2^	RMSE (stems/m^2^)	RRMSE (%)
Gréoux	0.92	32.04	0.83	24.67	6.54

Clermont	0.56	236.08	0.51	53.32	9.51

Gréoux & Clermont	0.76	111.00	0.80	45.73	9.22

**Table 7:** Biomass regression models derived from stem density, ear density, stem area, height, and biovolume at the Gréoux experimental site. Note: *∗∗* means model significant at the 0.01 level of probability. The R^2^, RMSE, and RRMSE are averaged R^2^, RMSE, and RRMSE values of leave-one-out cross-validation methods.

Variables	R^2^	RMSE (g/m^2^)	RRMSE (%)
Stem density	0.43*∗∗*	94	8.0

Ear density	0.51*∗∗*	84	8.1

Plant height	0.44*∗∗*	110	9.8

Basal area	0.64*∗∗*	77	7.3

Biovolume	0.81*∗∗*	62	5.8

All	0.85*∗∗*	58	5.1
